# Measuring and preventing alcohol use and related harm among young people in Asian countries: a thematic review

**DOI:** 10.1186/s41256-018-0070-2

**Published:** 2018-05-09

**Authors:** Heng Jiang, Xiaojun Xiang, Wei Hao, Robin Room, Xiaojie Zhang, Xuyi Wang

**Affiliations:** 1 Centre for Alcohol Policy Research, School of Psychology and Public Health, La Trobe University, Melbourne, VIC 3086 Australia; 2 Melbourne School of Population and Global Health, the University of Melbourne, Melbourne, VIC 3000 Australia; 3 Mental Health Institute of the Second Xiangya Hospital, Central South University, The China National Clinical Research Center for Mental Health Disorders, National Technology Institute of Psychiatry, Key Laboratory of Psychiatry and Mental Health of Hunan Province, Changsha, 410011 Hunan China; 4 Centre for Social Research on Alcohol and Drugs, Stockholm University, SE-106 91 Stockholm, Sweden

**Keywords:** Alcohol use, Alcohol related harm, Young people, Alcohol policy, Asia

## Abstract

**Background:**

The paper reviews alcohol consumption patterns and alcohol-related social and health issues among 15–29-year old young people in Asian countries, and discusses strategies for preventing and controlling alcohol use and related harms.

**Methods:**

We searched Google Scholar, PubMed, and Web of Science for reports, reviews and journal articles published in English between 1st Jan 1990 and 31st August 2016.

**Results:**

Forty-one reports, reviews and journal papers were identified and included in the final review. The current drinking levels and prevalence among young people are markedly different between eight included Asian countries, ranging from 4.2% in Malaysia to 49.3% in China. In a majority of the selected Asian countries, over 15% of total deaths among young men and 6% among young women aged 15–29 years are attributable to alcohol use. Alcohol use among young people is associated with a number of harms, including stress, family violence, injuries, suicide, and sexual and other risky behaviours. Alcohol policies, such as controlling sales, social supply and marketing, setting up/raising a legal drinking age, adding health warning labels on alcohol containers, and developing a surveillance system to monitor drinking pattern and risky drinking behaviour, could be potential means to reduce harmful use of alcohol and related harm among young people in Asia.

**Conclusions:**

The review reveals that drinking patterns and behaviours vary across eight selected Asian countries due to culture, policies and regional variations. The research evidence holds substantial policy implications for harm reduction on alcohol drinking among young people in Asian countries -- especially for China, which has almost no alcohol control policies at present.

## Background

Alcohol is a psychoactive substance with dependence-producing properties. Alcohol use is one of the leading risk factors for disease, disability and death among young and middle-aged men and women in many countries, associated with more than 200 diseases and injury conditions and with 2.3 million deaths attributed to it globally in 2015 [1, 2].

Asia is the fastest growing alcohol market, accounting for over 30% of global alcohol sales in 2014, with an estimated overall 176% growth from 2000 to 2019; China and India are leading the growth with rates of 382% and 1245% respectively [3]. Alcohol companies focus on youth drinking because they want to establish drinking habits that will be carried on and even expanded in middle age. Furthermore, binge drinking is more common among young people compared with older age groups [4, 5], and young persons are being targeted by alcohol marketers through the development of new products and sophisticated marketing techniques [6]. Motivations to drink in young people, which have been extensively studied [7, 8], commonly include to obtain social rewards such as camaraderie and approval, to enhance positive mood or wellbeing, to cope with negative emotions, and to avoid social rejection [9]. Nowadays, the internet and other social media have substantially extended the range of peer engagement, and gaining greater respect from peers is a major reason why youth is a risk period for alcohol use and abuse [10].

Although harmful use of alcohol among young people has been widely discussed in western countries [11], little attention has been paid in Asian countries. A recent World Health Organization (WHO) report highlighted the increasing adverse effects of alcohol use particularly on young people compared to older adults in still-developing Asian countries [8], pointing out that alcohol use among this group deserves special attention due to their biological and psychological vulnerability [8]. The present study reviews alcohol consumption patterns and related health and social issues among young people in Asian countries, defining young people as those aged 15–29. Strategies for prevention and control of alcohol-related harms are discussed and assessed in terms of their potential contribution to future policies to reduce harmful use of alcohol among young people in Asia.

## Search strategy

We conducted a comprehensive thematic review of PubMed, Web of Science and Google Scholar for reports, reviews and journal articles published in English between 1st Jan 1990 and 31st August 2016, with search terms pertaining to “alcohol use or alcohol consumption or drinking”, “current drinking or risky drinking or risk alcohol use or risky alcohol consumption” “young people or youth or adolescent or adolescence” “health outcome or social outcome or harm” and “Asia or Asian countries”. The full search method of the review is elaborated in the Appendix.

**Fig. 1:**
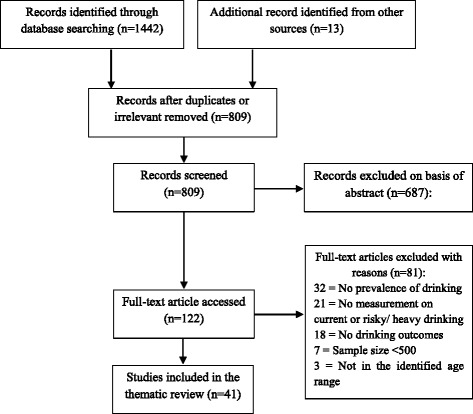
A flow diagram of article reviewed

Only three WHO reports [8, 12, 13] on youth drinking in Asia were found in the initial search. We then narratively searched “youth drinking or drinking among young people” in eight selected Asian countries to identify country-specific studies, including China, India, Japan, South Korea, Malaysia, Mongolia, Thailand and Vietnam. Because Asia contains a variety of cultures and civilisations, with different traditions of drinking, these eight countries have been chosen as countries of substantial size for which there is available data that will give a sense of patterns and the extent of variation in the region. Forty-one reports, reviews and journal papers were identified and included in the final review (see Fig. 1). We also reviewed several major international sources of data and information regarding alcohol use in young people or youth: WHO’s Global Information System on Alcohol and Health; WHO’s Global Status Report on Alcohol and Health 2014; the WHO STEPwise approach to Surveillance (STEPS) program; the Global School-based Student Health Survey (GSHS); the 2015 Annual World Drug Report published by UN’s Office on Drug and Crime; and the 2015 Global Burden of Disease Study.

The 2014/15 Survey of Drug Use among Students in Hong Kong and National Family Health Survey 2005–06 (NFHS-3) in India were used in this review as sources of the prevalence of current drinking among young people in Hong Kong and India. The GSHS is a longitudinal survey conducted in many countries, and the results from the latest survey in selected Asian countries were abstracted. We also reported some results from the 2015 Global Burden of Disease Study’s modelled estimates of number of deaths and percentage of total death attributable to alcohol use among young men and women aged 15–29 years in the eight Asian countries [1].

The measures -- of current drinking (in the past 30 days) or risky or heavy episodic drinking (60 g or more of alcohol consumed in a single drinking occasion in the last 12 months) or alcohol’s health or social harms among young people aged 15–29 years -- were abstracted from the reviewed studies. The Oxford 2011 Levels of evidence developed by the Oxford Centre for Evidence-Based Medicine [14] was used to assess the quality of studies in the thematic review.

## Prevalence and patterns of drinking among young people in Asian countries

### Prevalence of drinking and per capita alcohol consumption

**Fig. 2:**
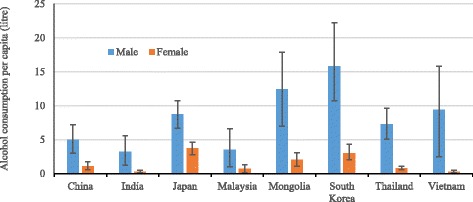
Alcohol consumption among males and females aged 15–19 in eight Asian countries in 2010. Alcohol consumption per capita (litres) among male and female 15–19-year olds; countries include China, India, Japan, Malaysia, Mongolia, South Korea, Thailand and Vietnam; 95% Confidence Interval bars are shown; alcohol consumption data in eight Asian countries and elsewhere are available in the WHO’s Global Information System on Alcohol and Health [15]

Among teenagers within Asia, there are big differences by country in the consumption of alcohol. Figure 2 shows alcohol per capita consumption among male and female youth aged 15–19 years in eight Asian countries in 2010 using data from the WHO’s Global Information System on Alcohol and Health [15]. Alcohol per capita consumption for male adolescents was higher than for females in all eight countries, and males aged 15–19 years in South Korea consumed substantially more alcohol per capita than male adolescents in China, India, Japan, Malaysia and Thailand.

**Fig. 3:**
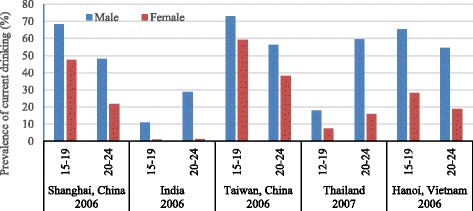
The prevalence of current drinking among males and females aged 12–24 years in five Asian regions. Prevalence of current alcohol consumption in the last 30 days (current drinking) among males and females 12–24-year olds; countries/regions include Shanghai (China), India, Taiwan (China), Thailand and Hanoi (Vietnam)

**Fig. 4:**
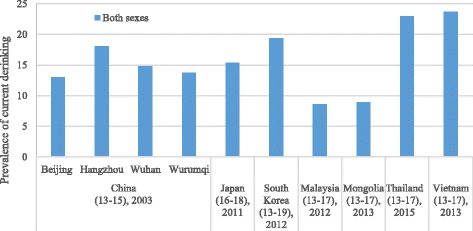
The prevalence of current drinking among youth aged 13–19 in selected Asian countries and districts. The prevalence of current drinking among youth both sexes 13–19-year olds in selected Asian countries and districts; the results were abstracted from Global School-based Student Health Surveys [66]

The prevalence of current drinking among young people has been measured in a wide variety of surveys in Asia (shown in Table 3 in the Appendix). There are 9 surveys reporting results for an age spread in the range of 15–24 years, and 8 more surveys where the lowest age in the spread was under 12 or the highest was above 29. However, many samples were of parts rather than the whole of the country and the reported rates often varied substantially between different surveys in the same country, even when the same age group was interviewed. Because of the inconsistency of survey methods and samples across different Asian countries, only five Asian countries for which data was available in the same age group were selected to compare the prevalence of current drinking among young males and females aged 15–24 years in Fig. 3. The figure shows that the prevalence of current drinking among 15–24-year old males and females in cities in China, Taiwan, and Vietnam was higher than in India and Thailand. Higher prevalence of current drinking was found in the older age group (20–24 years) in India and Thailand, which is opposite to the pattern in the other three countries or regions. The studies from the GSHS mainly focused on youth aged 13–19, and were conducted in different years across selected Asian countries (see Fig. 4), suggesting more than 10% of youth aged 13–19 were current drinkers in a majority of Asian countries.

### Heavy drinking

Heavy and risky drinking was common among young people, and those under age 35 had the highest levels of risky drinking practices in a study in Mongolia [16]. Among school students aged 12–17 in China, 11.4% of males and 5.1% of females had binge drinking at least once a month [17]. In South Korea, 7.9% of young males and 7.3% of young females had drunk heavily on 1–2 days in the past 30 days, while 1.6% of males and 0.9% of females had drunk heavily on more than 5 days in the past 30 days [18]. About 27% of teenagers aged 13–17 years and 26% of young adults aged 18–29 years had engaged in risky drinking in Malaysia [19]. In high schools in Japan, 3.0% of students were frequent drinkers (drank alcohol 10 days or more in the past month) [20]. In Thailand, very high-risk drinking (drinking more than 100 g for males and 60 g for females per day) was reported by 8.1% of boys and 1.8% of girls aged 12–19 years, and 16.6% of males and 4.6% of females aged 20–24 years. In a rural district in northern Vietnam, 35% of young people (18–24 years) were excessive drinkers, with 1.9% binge drinkers and 11.8% problem drinkers as measured by their AUDIT score [21]. In India, 59% and 21% of males and females aged 18–34 years drank heavily (over 60 g alcohol) in a single drinking occasion in the last 12 months, while the heavy episodic drinking rate was still higher in Japan -- 76% among young males and 45% among young females [22].

## Alcohol-related harm among young people in Asia

Adolescents and young people are typically more vulnerable to alcohol-related harm from a given volume of alcohol than other age groups [23–25]. Drinking and heavy drinking are also associated with a variety of social and health harms among young people in Asia, and may be instrumental in the occurrence of the harm. A summary of detailed studies of the relation of drinking to health and social harms among young people from survey studies in eight Asian countries is presented in Tables 4 and 5 in the Appendix. Heavy drinking was found to be significantly associated with suicidal ideation, suicidal attempts and, self-distress in many Asian countries [19]. HIV risk behaviours have been found to be strongly linked with binge drinking and its frequency among rural adolescents in Vietnam [26]. Effective measures to prevent binge drinking are thus essential to HIV prevention, especially among youth aged 18–20 years [26, 27]. Students who started drinking at age 12 or younger, and at 13 or 14 years, were significantly more likely to be currently smoking, currently drinking, engaging in heavy smoking and heavy drinking than students who began drinking at age 15 or older. Studies from Western countries suggest that before 14 years of age, early onset of alcohol use was a predictor of impaired health status because it was associated with increased risk for alcohol dependence and abuse at later ages, alcohol-related motor vehicle crashes, and other unintentional injuries [27–35]. Adolescents who have used alcohol were more likely to have used other substances, and to have engaged in sexual behaviours and fights [36], while young people with current alcohol consumption were more likely to have poor academic performance, family conflict or social problems [37].

The projections in the recent Global Burden of Disease Study 2015 (Table 1) found that alcohol was ranked as the greatest risk factor for young and middle aged men in most Asian countries (but not in Malaysia), both in terms of attributable disability-adjusted life-years lost and mortality rates [1]. More than 15 % of total deaths among young men aged 15–29 years were attributable to alcohol use in China, Japan, South Korea, Thailand and Vietnam (see Table 1), amounting to a large number of deaths each year. The proportion of total deaths attributable to alcohol use was smaller in India and Malaysia. South Korea had the highest percentage of deaths attributed to alcohol use among both males and females.

**Table 1:** Number and percentage of deaths attributable to alcohol use among males and females aged 15–29 years in selected Asian countries in 2015 (data from the Global Burden of Disease Study 2015 [1])

Country	Men			Women		
	15–19 years	20–24 years	25–29 years	15–19 years	20–24 years	25–29 years
China	4313 (16.6%)	10,117 (20.3%)	16,518 (20.6%)	902 (8.8%)	1624 (8.9%)	2314 (7.7%)
India	4752 (6.0%)	10,148 (8.2%)	15,317 (10.2%)	1905 (2.5%)	2353 (2.4%)	2814 (2.8%)
Japan	152 (17.5%)	314 (18.5%)	335 (16.2%)	44 (9.9%)	84 (11.6%)	103 (10.7%)
Malaysia	82 (6.7%)	164 (8.1%)	176 (7.0%)	13 (2.6%)	16 (2.6%)	16 (2.1%)
Mongolia	20 (12.5%)	62 (18.5%)	115 (22.4%)	4 (6.5%)	8 (6.5%)	15 (8.8%)
South Korea	117 (20.6%)	201 (22.1%)	253 (21.3%)	30 (11%)	66 (13.9%)	77 (12.9%)
Thailand	390 (17.7%)	819 (20.9%)	1147 (20.0%)	77 (9.5%)	113 (8.9%)	148 (7.5%)
Vietnam	559 (14.7%)	1278 (17.9%)	1462 (17.7%)	89 (7.8%)	134 (7.2%)	141 (6.3%)

## Strategies for prevention and control

Despite the large health and social burden associated with harmful use of alcohol, it has remained a relatively low priority in public health policy in most Asian countries [38]. Harmful use of alcohol can have serious health, social and economic consequence both for the drinker and for others around the drinker, and for society at large. Population-based interventions have been considered as effective means to reduce alcohol consumption and alcohol-related harm. The WHO has recommended five “best buys” – the most cost-effective policy interventions to tackle harmful use of alcohol in the national context, including restricting access to retail alcohol, raising taxes/prices on alcohol, imposing bans on alcohol advertising, enforcing drink driving laws, and offering brief advice for hazardous drinking [39]. We compared alcohol policies within these five “best buys” across the eight Asian countries, using policy data from the World Alcohol Strategy 2014 [40] (shown in Table 2). It should be recognised that some policies are set at subnational levels, and may vary within a country; this is particularly true for India. There is a wide range in Asia in terms of the extent of market and behavioural regulation around alcohol; it is apparent that “alcohol policy” has not yet really hit China in the era when a freer entrepreneurial market makes it a public health necessity.

**Table 2:** Comparison of “WHO’s five best buys” alcohol policy interventions among eight Asia countries in 2014 [40]

Policies and interventions			China	India	Japan	South Korea	Malaysia	Mongolia	Thailand	Vietnam
Restrict access to retailed alcohol	Minimum drinking age		✘	21 or higher in most states	20	19	18	21	20	18
	Restrictions for on−/off-premise sales	Hours/days	✘ / ✘	Sub national	✘ / ✘	✘ / ✘	✔ / ✘	✔ / ✔	✔ / ✔	✘ / ✘
		Places/density	✔ / ✔	Sub national	✘ / ✘	✘ / ✘	✔ / ✔	✔ / ✔	✔/ ✘	✘ / ✘
Raise taxes/prices on alcohol	Alcohol excise or tax		✔	✔	✔	✔	✔	✔	✔	✔
	Legally binding alcohol sales promotion / sponsorship		✘/ ✘	✔ / ✔	✘ / ✘	✘ / ✘	✘ / ✘	✔ / ✘	✔ / ✔	✘ / ✘
Enforce bans on alcohol advertising	Advertising / product placement		✘ / ✘	✔ / ✔	✔ / ✔	✔ / ✘	✔ / ✔	✔ / ✘	✔ / ✔	✔ / ✔
Enforce drink driving laws	Sobriety check-points		✘	✘	✔	✔	✔	✘	✔	✔
	RBT / BAC level		✔ / 0.02	✔ / 0.03	✘ / 0.03	✘ / 0.05	✔ / 0.08	✔ / 0.05	✘ / 0.05	✘ / 0.00
Offer brief advice for hazardous drinking	Number of standard drinks		✘	✘	✘	✘	✘	✘	✔	✔
	Alcohol content displayed on containers		✔	✔	✔	✔	✔	✔	✔	✔
	Health warning labels on advertisements / container		✘ / ✘	✔ / ✔	✘ / ✘	✘ / ✔	✘ / ✘	✘ / ✘	✔ / ✔	✘ / ✘

“✘” is no such policy and “✔” is yes. “Best buys” are WHO recommended the most cost-effective alcohol policy interventions to tackle harmful use of alcohol

The majority of Indian states have set their minimum ages at 21 years or higher [41], resulting in relatively low prevalence of alcohol drinking among young people [42]. In contrast, there is a substantially higher prevalence of alcohol consumption among young people aged 16–29 years in China (see Table 3), which is the only country among the eight Asian countries in Table 1 without a law for a minimum legal drinking age. Compared with six other Asian countries, Mongolia is the only country which has a national alcohol monopoly and there are state level alcohol monopolies in some Indian states [40, 43]. Government monopolies for the sale of alcohol could reduce alcohol consumption and related harm among young people, and such a system allows for more limits on the number of liquor stores and the trading hours and days than the private sale system [44, 45]. Nevertheless, restricting availability may have much less effect in countries where one third of the alcohol drunk is informally produced and sold outside any government control or regulation. For example, unrecorded alcohol consumption per capita in India was estimated at 2.2 l in 2010, while recorded alcohol consumption was 4.3 l [15].

Increasing alcohol prices delays the start of drinking, slows young people’s progression towards drinking large amounts, and reduces young people’s heavy and risky drinking behaviour [46, 47]. Table 2 shows that alcohol tax policies exist in all selected Asian countries. A study in Vietnam found increasing the price and reducing the availability of alcohol are potential means to reduce alcohol use among young people [48]. The taxation system in Thailand, ‘Two-Chosen-One’ (2C1), combines specific taxation (as a function of the alcohol content) and ad valorem taxation (as a function of the price); the higher one will be chosen when taxes are calculated according to the two alternatives, which it is argued results in a great potential to reduce simultaneously alcohol consumption and prevent drinking initiation among young people [49].

Alcohol is often supplied to adolescents and young adults by their parents, care-givers and peer friends. In China and South Korea, adolescents were often introduced to alcohol at home or friend’s home via a taste from their parents’ liquors during family gatherings or special celebration events [8, 50]. Protective parenting practices and disapproving caregiver’s attitudes towards youthful alcohol use can deter alcohol use among young people [51]. Social supply of alcohol to adolescents should be regulated via legislation, as early drinking leads to higher alcohol dependence in later life. Asian countries can learn from the social supply laws in Australia, which provide that supplying alcohol to a minor is illegal [52].

Evidence from longitudinal studies suggests that initiation of youth drinking and of riskier patterns of youth drinking are affected by exposure to alcohol advertising in social media, or in the form of movie content or of alcohol-branded merchandise [53], and similar associations were found in Taiwan, China [54]. The effects of exposure to alcohol advertising seem to be cumulative; young people are likely to continue increasing their consumption as they move into their mid-20s in markets with a greater amount of alcohol advertising [55]. Some Asian countries (such as China, Thailand and Vietnam) have implemented a partial ban of alcohol advertising in some media. Others have not had any alcohol advertising regulations (e.g. Japan and Laos). Alcohol companies are sponsors of major music and sports events where youth are heavily involved, sponsoring the International Music Summit in China, the Japanese team in the Olympics, Thai football teams and World Cup soccer [56]. In contrast, direct alcohol advertising is not permitted in the broadcast media or on billboards in Malaysia, except in the state of Sabah; this reduces exposure to alcohol advertisements and may prevent alcohol-related harms among young people [57]. Bans or restrictions on alcohol advertising and sponsorship have been called for by public health researchers and bodies, because in some Asian countries (e.g., China and Japan), alcohol brands promote their products using sport and movie stars or successful business men, which shapes young people’s drinking attitude and behaviour.

School-, family- and community-based interventions have been found effective in reducing harmful use of alcohol among young people [58–60]. However, most of these interventions have been carried out in western developed countries. High quality interventions that target on youth drinking and other risky behaviours are recommended to be applied and evaluated in low- and middle-income countries, particularly in Asia.

### Limitations

It is worth noting that a number of Asian studies on youth drinking, harms and alcohol policies were published in local (non-English) languages and were unable to be captured in this study. Another limit is that some of the findings used in the discussions of strategies for prevention come from non-Asian countries.

## Conclusion

Harmful use of alcohol has been one of the global public health challenges among young people in the last decade, particularly in Asian countries. Over 70% of the total population of the earth lives in Asia, and Asian developing countries such as India, China, Thailand and Vietnam have been targeted by global alcohol corporations as emerging alcohol markets in recent decades. Alcohol imports in the developing Asian countries, such as Thailand and China, have been increasing significantly, facilitated by free trade agreements coming into effect in recent years [61, 62]. The potential adverse public health effects from alcohol, tobacco and other drugs need to be taken into account in negotiating new international trading agreements involving Asian countries. More attention should be paid to the potential adverse effects of limiting governments’ ability to control the alcohol market in increasing rates of alcohol problems, particularly among youth and young adults. To the best of our knowledge, there are as yet no formal studies of the social and economic costs of harmful use of alcohol among young people in Asian countries.

This review suggest that more coherent and focused research efforts are urgently needed on youth drinking and its problems in Asia, where drinking is rising so rapidly. Public health research and policymaking needs to catch up with the alcohol industry’s realisation that what happens in a youth cohort strongly affects their market for the next half-century. Studies on social and economic harm and cost of drinking among young people in Asia could be a good step forward, incorporating both population surveys and health, police and welfare register data. A recent collaborative project [63] (Alcohol’s Harm to Other in five Asian countries) funded by the WHO and Thai Health Promotion Foundation may help to fill this research gap.

Prevention of the health and social issues related to youth drinking will be a major goal for public health in Asian countries that follow WHO strategies to prevent non-communicable diseases and road-traffic injuries by reducing harmful alcohol use within the national context [64, 65]. Establishing or improving national monitoring and surveillance systems to inform alcohol policy in Asian countries could be the first step. But what is needed beyond this is a multi-level approach that integrates policy actions (e.g., sales, social supply and marketing), regulations (e.g., drinking age laws), health education (e.g., media campaigns and health warning labels), and effective health system responses (brief intervention, and alcohol abuse and dependence treatment). This is an urgent mission for public health action in all Asian nations. The review has particular policy implications for the Chinese government and health authorities in tackling the toll of death and disease among young people, given that China at present has almost no alcohol control policies.

## Appendix

### Search method of the systematic review

We conducted a comprehensive thematic review of PubMed, Web of Science and Google Scholar for reports, reviews and journal articles published in English between 1st Jan 1990 and 31st August 2016, with search terms pertaining to “alcohol use or alcohol consumption or drinking”, “current drinking or risky drinking or risk alcohol use or risky alcohol consumption” “young people or youth or adolescent or adolescence” “health outcome or social outcome or harm” and “Asia or Asian countries”.

Articles returned for those mentioned search terms were assessed for relevance. Studies were excluded if their abstracts or titles did not refer to the possibility of an association between alcohol or drinking and youth or young people or adolescent or adolescence and Asia or Asian countries. The final review was conducted on full texts of paper meeting this criterion. The initial search and article review were conducted in July 2016 by X.X., and the second search and review were conducted in September and October 2016 by H.J.

In Google Scholar, the search terms gave more than 16,000 potential records, and the first 500 of these (sorted by relevance) were examined by title and abstract, and 60 items selected for further inspection. Studies were included in the final review if they included measures of current (past 30-day drinking) or risky or heavy episodic drinking (drink 60 g alcohol or more in a single drinking occasion in the last 12 months) or alcohol’s health or social outcomes or harms among young people aged 15–29 years. In the initial search we did not find any journal articles discussed youth drinking or drinking among young people in Asia and only three WHO reports [12–14] were found related to the topic. We then decided to narrow our search scope to eight selected Asian countries, including China, India, Japan, South Korea, Malaysia, Mongolia, Thailand and Vietnam. Because Asia contains a variety of cultures and civilisations, with different traditions of drinking, these eight countries have been chosen as countries of substantial size for which there is available data that will give a sense of patterns and the extent of variation in the region. Forty-one reports, reviews and journal papers were identified and included in the final review.

In the review process, we found many studies were conducted based on a small sample size without any justification of statistical power, response rates, random or non-random selection and sample representativeness. We found studies with sample size larger than 500 generally provided more reliable measurement on youth drinking behavior and related harms. Studies with sample sizes smaller than 500 were excluded. Studies that only measured age of initial drinking or frequency of heavy drinking or alcohol abuse or alcohol dependence or knowledge, attitudes, and practices related to alcohol use were excluded. Alcohol consumption data reported in the most recent country-level national survey reports were collected. We also reviewed several major international sources of data and information regarding alcohol use in young people or youth: WHO’s Global Information System on Alcohol and Health; WHO’s Global Status Report on Alcohol and Health 2014; the WHO STEPwise approach to Surveillance (STEPS) grogram; the Global School-based Student Health Survey (GSHS); the 2015 Annual World Drug Report published by UN’s Office on Drug and Crime; and the 2015 Global Burden of Disease Study.

The measures -- of current drinking (in the past 30 days) or risky or heavy episodic drinking (60 g or more of alcohol consumed in a single drinking occasion in the last 12 months) or alcohol’s health or social harms among young people aged 15–29 years -- were abstracted from the reviewed studies. The Oxford 2011 levels of evidence developed by the Oxford Centre for Evidence-Based Medicine [15] was used to assess the quality of studies in the thematic review. Identified published reports, reviews or journal papers were obtained and reviewed by X.X. and H.J. and data were extracted from these articles. Data were sought on countries and year of data collection.

An example of search approach for alcohol consumption among young people in eight Asian countries in **PubMed** is,

(((youth) OR young) OR adolescent) OR adolescence.

AND (((alcohol) OR drinking) OR ethanol).

AND ((((((((China) OR India) OR Japan) OR Korea) OR Malaysia) OR Mongolia) OR Thailand) OR Vietnam).

AND (“1990/01/01”[PDAT]: “2016/08/31”[PDAT]).

AND “humans”[MeSH Terms].

AND English[lang].

After the initial search in PubMed, 5636 studies were found in English. Further checking the titles and abstracts, the number of studies was narrowed to 373.

A search approach for alcohol consumption among young people in eight Asian countries in **Web of Science** is,

(Alcohol* OR drinking* OR ethanol*) AND TOPIC: (young* or youth* or adoles*) AND TOPIC: (China* OR India* OR Japan* OR Korea* OR Malaysia* OR Mongolia* OR Thailand* OR Vietnam*)

Refined by: LANGUAGES: (English) AND DOCUMENT TYPES: (Article OR Review OR Book chapter OR Report) AND Timespan: 1990–2016.

After the initial search in Web of Science, 2142 studies were found. After further quick scanning the titles and abstracts, number of studies were narrowed to 569.

### Search engines used in this study

Three powerful academic search engines were used in this review: PubMed, Web of Science and Google Scholar. The commonly used databases in social and medical research, such as Medline, Social Science Index, and BIOSIS Citation Index are already included in the PubMed and Web of Science search databases. Please see Medline resource guideline https://www.nlm.nih.gov/bsd/pmresources.html and Web of Science database collection https://clarivate.com/products/web-of-science/databases/

**Table 3:** The prevalence of current drinking among young people in eight Asian countries

Country/region & year	Authors	Sample size	Levels of evidence^g^	Age (years)	Prevalence of current drinking (%)		
					Male	Female	All
WHO Global School-based Student Health Survey (GSHS)							
China, Beijing, 2003	GSHS [66]	2348	1	13–15	17.7	8.6	13.0
China, Hangzhou, 2003	GSHS [66]	1802	1	13–15	20.9	15.0	18.1
China, Wuhan, 2003	GSHS [66]	1947	1	13–15	19.7	9.7	14.8
China, Wurumqi, 2003	GSHS [66]	2918	1	13–15	16.3	11.0	13.7
Malaysia, 2012	GSHS [66]	25,507	1	13–17	11.0	6.3	8.6
Mongolia, 2013	GSHS [66]	5393	1	13–17	10.8	7.1	8.9
Thailand, 2015	GSHS [66]	5894	1	13–17	27.2	19.2	23.0
Vietnam, 2013	GSHS [66]	3331	1	13–17	31.3	16.5	23.7
Other identified studies							
China, Taiwan, 1994–96	Lin et al. [67]	4463	3	17–20	–	–	24.6
China, Beijing and Shanghai, 2001–02	Cheng et al., [68]	1209	3	18–34	–	–	35.0
China, Taipei, 2006	Zabin et al., [69]	4354	3	15–24	73.0^c^	59.3^c^	–
					56.2^e^	38.1^e^	
China, Shanghai, 2006	Zabin et al., [69].	6299	3	15–24	68.4^c^	47.5^c^	–
					48.1^e^	21.9^e^	
China, 2009	Ji et al., [70].	52,150	1	16–29	66.6	34.7	49.3
China, Hong Kong, 2014–15	Security Bureau of HK [71]	95,788	1	10–21	21.2	19.2	20.2
India, 1996–97	Mohan et al., [42].	7037	3	15–29	–	–	8.9^c^ 24.8^f^
India, 2003	Wilsnack et al., [22] (GENACIS study)	1641	3	18–34	33.8	1.8	–
India, 2006	Parasuraman et al., [72]	73,062	1	15–24	11.0^c^	1.0^c^	–
					28.8^e^	1.4^e^	
Japan, 1999	Takaura and Wake [20]	1466	3	9–17	41.8	35.1	38.4
Japan, 2001	Wilsnack et al. [22] (GENACIS study)	575	2	18–34	93.9	88.3	–
Japan, 2008	Takakura [73]	3248	3	15–18	19.9	20.2	–
Japan, 2011	WHO [8]	9778	1	16–18	14.7	16.0	15.4
South Korea, 2006	Han et al. [18]	70,486	1	12–19	11.0	9.1	10.4
South Korea, 2012	WHO [8]	80,000	1	13–19	22.7	15.8	19.4
Malaysia, 2011	Mutalip et al. [19].	21,011	2	13–29	–	–	4.2^b^ 14.0^d^
Malaysia, 2012	Yusoff et al. [74]	25,507	2	12–18	–	–	7.5
Mongolia, 2009	WHO [75]	899	1	15–24	–	–	26.7
Mongolia, 2010	Demaio et al. [16]	1100	3	15–24	–	–	22.0
Thailand, 2007	Assanangkornch et al. [76]	6380	3	12–24	17.9^a^	7.3^a^	12.7
					59.5^e^	15.8^e^	
Thailand 2007–08	Pichainarong and Chaveepojnkamjorn [77]	11,360	2	10–21	12.2	5.8	
Thailand, 2010–11	Lohsoonthorn et al. [78]	2854	3	18–20	–	–	34.2
Vietnam, Hanoi, 2006	Zabin et al. [69]	6363	3	15–24	65.4^c^	28.3^c^	–
					54.6^e^	18.8^e^	

^a^12–19 years old; ^b^ 13–17 years old; ^c^ 15–19 years old; ^d^18–29 years old; ^e^ 20–24 years old; ^f^ 20–29 years old; GENACIS is the Gender, Alcohol and Culture: An International Study

^g^The Oxford 2011 Levels of evidence developed by the Oxford Centre for Evidence-Based Medicine [14] was used to assess the quality of studies in the thematic review

**Table 4:** Harm experienced related to alcohol use among young people in eight Asian countries

Country & authors	Study type & year	Age	Sample size	Harm experiences
China				
Wan et al., 2011 [79]	Cross-sectional survey in 6 cities	12–24	17,622	Alcohol use was significantly positively correlated with deliberate self-harm behaviors among youth.
Zhou et al., [35]	cross-sectional survey in Shenzhen City	10–24	4138	None-fatal injuries among young people were significantly correlated with alcohol consumption in the last 12 months
India				
Esser et al., 2015 [80]	Household survey interview in 5 cities	15–24	741	About 23% females and 24% males aged 15–24 years reported experienced 5 or more types of harms due to other’s drinking, including physical, sexual, psychological, financial and social harms.
Madhivanan et al., 2014 [81]	Cross-sectional survey in Mysore, 2005–06	15–30	898	Alcohol use was significantly associated the prevelance of intimate partner physical violence.
Nadkarni et al., 2015 [82]	Cross-sectional survey in Goa	16–24	3663	Current alcohol use was significantly associated with physical violence in both young men and women.
Japan				
Higuchi et al., 1994 [37]	Cross-sectional survey	18–29	1225	Drinking alcohol in the past 12 months was significantly associated with experience of social problems.
South Korea				
Han et al., 2009 [18]	Nationally representative survey	12–19	71,404	Heavy drinking were found to be significantly associated with suicidalideation and suicidal attempts among boys and girls
Kang et al., 2014 [83]	Sixth Korea Youth Risk Behavior Web-based Survey in 2010	12–18	72,623	Alcohol consumption was significantly associated with suicide ideation and attempt among adolescents.
Park et al., 2010 [84]	Household survey, 2005	18–39	5333,	Volume of alcohol consumption was significantly correlated with suicide ideation among young male and female adults.
Seo et al., 2015 [85]	Cross-sectional survey 2008–2011	19–30	1176	Alcohol consumption volume and frequency were significantly and negatively associated with bone mineral density in young women.
So and Park, 2016 [86]	Online self-administered questionnaire survey	12–18	74,186	Alcohol consumption was associated with youth low academic performance.
Malaysia				
Chan et al., 2013 [87]	cross-sectional survey 2008–09	17–18	4581	Alcohol use was significantly associated with youth suicidal behavior.
Manickam et al., 2014 [36]	cross-sectional school-based survey 2012	12–17	25,285	Adolescents who had used alcohol were more likely to have used substance, experienced injuries and engaged in sexual behaviours.
Mutalip et al., 2014 [19]	Cross-sectional population-based study 2011	12–29	746	Feeling of remorse/guilt after drinking; blackouts; injury to self or others and received abuse from others due to drinking.
Mongolia				
n/a	n/a	n/a	n/a	n/a
Thailand				
Assanangkornchai et al., 2009 [88]	Cross-sectional survey,	10–22	50,033	Alcohol use was significantly associated with a series of risk behaviours among youth, including drug misuse, drink-driving, injuries, physical violence, depression, suicide attempt and unwanted pregnancy.
Balogun et al., 2014 [89]	Self-administered school-based survey	12–15	2761	Past 30-day alcohol use and lifetime drunkenness were associated with depression and anxiety-induced sleeplessness among students.
Lohsoonthorn et al., 2013 [78]	Cross-sectional survey, 2010–11	18–22	2854	Alcohol consumption was significantly associated with increased odds of daytime dysfunction and sleepiness.
Nakahara et al., 2005 [90]	Epidemiology study, injury data from 1998 to 2002	20–29	4015	Alcohol use was a significant predictor of fatal motocycle injuries.
Tongklao et al., 2014 [91]	Cross-sectional survey, 2011–12	10–18	2267	Alcohol use before or during riding was significantly associated with motorcycle injuries and risky riding behaviours.
Vietnam				
Diep et al., 2010 [92]	Cross-sectional survey in two universities	18–29	619	12.3% of the total sample were identified with an AUDIT score of 8 or higher.
Diep et al., 2013 [93]	Survey intervew on university students	17–28	699	Among drinking students: 47% of them had experienced negative influence on daliy activities; 8.9% of them reported having had social conflict; 68% of them reported loss of control, acute consequences, withdrawal; 22% of them had a mental health condition and physical illness; 12% of them had a medical health problem.
Diep et al., 2015 [48]	Cross-sectional survey in 12 universities in 4 province in Vietnam	Mean age 20.1	6011	Students reported experience of physical and amenity harms, including sleep and study disturbances, property damage, being insulted/quarrelling, unwanted sex, being beaten/fighting/pushed/hit, traffic accident/crash due to other’s drinking.
MT et al., 2012 [94]	Two National Population-Based Surveys -- Survey Assessment of Vietnamese Youth (SAVY 1 & 2)	14–19	4609 in SAVY 1 6508 in SAVY 2	Alcohol use was significantly associated with experiences of low mood and suicidal behaviors
Tho et al. 2007 [95]	Cross-sectional survey in Nha Trang City	14–25	880	Young people who drink alcohol were more likely to be sexually active and to have had unsafe and unprotected sex.

n/a means no papers or studies were identified within selection critriea

**Table 5:** Harm experience among young people studied in eight Asian countries, ranked by the number of studies reporting the harm

Harm experience	Age and gender	Countries and number of studies (N)
Deliberate self-harm and suicidal behaviors	12–24, both sexes	China, Malaysia, South Korea, Thailand and Vietnam (7)
Physical violence	15–24, both sexes	India, Thailand, Vietnam (6)
Fatal and none-fatal injuries	10–24, both sexes	China, Malaysia, Thailand, Vietnam (5)
Risky sexual behaviour	15–24, both sexes	India, Malaysia, Thailand, Vietnam (5)
Psychological problem, depressed	15–24, both sexes	India, Thailand, Vietnam (4)
Financial and social harms	15–24, both sexes	India, Japan, Vietnam (3)
Poor academic performance/ study disturbances	12–18, both sexes	South Korea, Vietnam (2)
Low bone mineral density	19–30, female	South Korea (1)
Harm/injury to others	12–29, both sexes	Malaysia (1)
Drug misuse	10–22, both sexes	Thailand (1)
Drink driving	10–22, both sexes	Thailand (1)
Alcohol dependence	18–29, both sexes	Vietnam (1)
